# Supplementary material for: Gpufit: An open-source toolkit for GPU-accelerated curve fitting
Source: Sci Rep. 2017 Nov 16;7:15722. doi: 10.1038/s41598-017-15313-9 (PMC5691161; doi:10.1038/s41598-017-15313-9)
Supplement: Supplementary file 1 — Supplementary Information [file 41598_2017_15313_MOESM1_ESM.doc]

Gpufit: An open-source toolkit for GPU-accelerated curve fitting

Adrian Przybylski, Björn Thiel, Jan Keller-Findeisen, Bernd Stock, and Mark Bates

Supplementary Information

Table of Contents

[Calculating with CUDA 2](#__RefHeading___Toc489893664)

[Gpufit performance characterization 2](#__RefHeading___Toc489893665)

[Computer hardware 2](#__RefHeading___Toc489893666)

[Test software 3](#__RefHeading___Toc489893667)

[Test datasets 4](#__RefHeading___Toc489893668)

[Test parameters 5](#__RefHeading___Toc489893669)

[Test outputs 5](#__RefHeading___Toc489893670)

[STORM experiment 5](#__RefHeading___Toc489893671)

[Microscope and sample preparation 5](#__RefHeading___Toc489893672)

[Supplementary Figure S1: GPU processing example 6](#__RefHeading___Toc489893673)

[Supplementary Figure S2: Gpufit modular design 7](#__RefHeading___Toc489893674)

[Supplementary Figure S3: Gpufit vs. MINPACK comparison 8](#__RefHeading___Toc489893675)

[Supplementary Figure S4: Fit precision and accuracy 9](#__RefHeading___Toc489893676)

[Supplementary Figure S5: Cpufit program flow 10](#__RefHeading___Toc489893677)

[Supplementary Figure S6: Gpufit program flow 11](#__RefHeading___Toc489893678)

[Supplementary Figure S7: Precision of Gpufit and GPU-LMfit 12](#__RefHeading___Toc489893679)

[Supplementary Figure S8: Comparison of fit estimators 13](#__RefHeading___Toc489893680)

[Supplementary Table S1: Test parameters 14](#__RefHeading___Toc489893681)

[References 15](#__RefHeading___Toc489893682)

# Calculating with CUDA

In general, the Central Processing Unit (CPU) of a computer executes program code sequentially, and the speed of execution is set by the CPU clock rate. For algorithms which lend themselves to parallelization, higher performance may be achieved by a Graphics Processing Unit (GPU). The essential difference between a CPU and a GPU is the architecture, which in the GPU consists of many individual processors (multiprocessors) which run concurrently. Each multiprocessor of a GPU is organized into many computing blocks, and each block is capable of executing multiple program threads. The architecture of the GPU is parallelized at the multiprocessor, block, and thread level, as illustrated in Supplementary Fig. S1. Consequently, an operation which needs to be repeated $N$ times on a CPU can be accelerated by executing it in a single step using $N$ independent threads of a GPU.

CUDA is a computing platform which enables general purpose, parallel computing on Nvidia CUDA-enabled GPUs^1^. A simple example of a parallelized operation is a function which subtracts the values of one set of arrays (*arrays1*) from a second set of arrays (*arrays2*), as shown in Supplementary Fig. S1. In this example, the results of the calculation for all array elements are obtained simultaneously. This is facilitated by the organization of each multiprocessor into blocks and threads. Here, each array subtraction is assigned to one block, and the calculation of each element of the output array is executed by the individual threads within the block. The variables *block_index* and *thread_index* allows the code executing on each thread to know for which part of the computation it is responsible.

CUDA functions resemble extended C functions. The number of required threads must be defined by two parameters in the function call, enclosed in *<<< >>>*. The first parameter represents the number of thread blocks, and the second represents the number of threads per block. In the example shown in the figure, the number of blocks corresponds to the number of pairs of arrays to be subtracted and the number of threads corresponds to the number of elements in each array. Thread blocks are evenly distributed across the multiprocessors and are executed independently. The more processors a GPU has, the larger the number of thread blocks that may be executed concurrently.

# Gpufit performance characterization

## Computer hardware

All tests were executed on a PC running the Windows 7 64-bit operating system and Nvidia Graphics driver version 378.78. The PC hardware included an Intel Core i7 5820K CPU, running at 3.3 GHz, and 64 GB of RAM. The graphics card was an NVIDIA GeForce GTX 1080 GPU with 8 GB of GDDR5X onboard memory. To avoid fluctuations in the CPU processor speed during testing, Windows was configured in a “High Performance” mode via the Power Options window. Specifically, the “Minimum processor state” setting was set to 100%, ensuring that the CPU clock remained at its maximum speed.

## Test software

The Cpufit, Gpufit, and C/C++ Minpack libraries were compiled using Microsoft Visual Studio 2013, with compiler optimizations enabled (release mode). The CUDA Toolkit used to build the software was version 8.0. CMake was used to automatically configure and generate the Visual Studio solution files. Instructions for building the Gpufit library are included with the Gpufit documentation ([http://gpufit.readthedocs.io](http://gpufit.readthedocs.io/)).

Each performance test was written as a Matlab script, and calls to Gpufit, Cpufit, Minpack, and GPU-LMFit were made via Matlab external code interfaces (MEX files). For this purpose, either 32-bit or 64-bit Matlab was used (version R2015b for 32-bit processing, and R2016b for 64-bit processing). All tests were run in 64-bit mode, with the exception of any test involving the GPU-LMFit software, which was only available as a 32-bit compiled static library file (.lib). MINPACK fitting was accomplished by calling the function lmder() from the MINPACK library. Code profiling tests were made using special versions of Cpufit and Gpufit having built-in timing functions.

The binary files required to run GPU-LMFit were included with the publication describing the software.^2^ Specifically, a Visual Studio 2010 project “GPU2DGaussFit”, which provides an example of how to use GPU-LMFit for fitting of a 2D Gaussian function. We reduced this project to its essential components by removing operations such as the initial parameter estimation and background correction in order to allow a fair comparison with Gpufit. The project was compiled using Visual Studio 2010 and CUDA 8.0 to yield a MEX file. Fitting of 2D Gaussian functions with this package was accomplished using the compiled MEX file.

Integration of Gpufit with Picasso was accomplished by including the pyGpufit module into the Picasso source code. Picasso was downloaded from the publicly accessible Git repository <http://github.com/jungmannlab/picasso>. An option was added to the user interface to allow the selection of the Gpufit function for curve fitting. When this option was selected, Picasso used Gpufit instead of its original multi-threaded curve fitting code. The fit tolerance and maximum number of fit iterations used for the call to Gpufit were the same as for the CPU-based Picasso fitting. The code was executed using the Python 3.5 interpreter, running in 64-bit mode on the same PC as described above. Additional timing functions were added to the python code in order to measure the actual execution time of the fit process, on either the CPU or the GPU. The source code for the initial version of Gpufit-modified Picasso is available at <http://github.com/gpufit/picasso/tree/gpufit_integration_initial>.

## Test datasets

Simulated datasets were generated in order to test the curve fitting algorithms. For this data, we simulated 2D Gaussian functions, according to the following expression:

$$f \left(x,y\right) = A \text{exp} \left[\frac{\left(x-x_{0}\right)^{2}+\left(y-y_{0}\right)^{2}}{2\sigma^{2}}\right] + b$$

where $\left(x_{0},y_{0}\right)$ are the center coordinates of the Gaussian peak, $A$ is the amplitude, $\sigma$ is the peak width, and $b$ is the constant baseline level.

The size of each dataset was characterized by the number of data points per fit (defined by the size $L$ of a square region, such that the total number of points per fit is $L^{2}$). The total number of fits per dataset was defined as $N$. The number of fits per dataset $N$ was varied between 1 fit and 1x10^8^ fits, and the fit size $L$ was varied between 5 and 25.

For each test data point, the parameters of the simulated Gaussian peaks were held constant with the exception of the center coordinates $\left(x_{0},y_{0}\right)$ which were randomly generated. The range of the center coordinates was defined by the mean center position $\left(x_{0},y_{0}\right)$ and a maximum deviation $\left(\Delta x_{\text{max}},\Delta y_{\text{max}}\right)$. For all tests, the mean center position was set equal to the center of the fit region, and the deviation was selected from a uniform distribution.

The initial guesses for all fits calculated with Cpufit, Gpufit, Minpack, and GPU-LMFit were randomized, within a specified range of the true value. The maximum offset for each parameter was calculated according to a maximum offset fraction $f_{\text{offset}}$ multiplied by the mean value of the true fit parameter. For example, if the true value of the Gaussian amplitude was 500 and $f_{\text{offset}}$ was set to equal 0.3, then the maximum random offset for the initial guess of the amplitude was 150. In this case, the initial guess for the amplitude would be chosen from a uniform distribution ranging between 350 and 650. This method was applied to the initial guesses for each fit parameter. For the center coordinate parameters, the maximum offset was calculated by multiplying $f_{\text{offset}}$ by the Gaussian width $\sigma$, in order to avoid any dependence of the results on the size of the fit area.

Noise was added to the data as either Gaussian-distributed noise or Poisson-distributed noise. For the case of Poisson noise, after calculating the simulated input data, each data point was replaced with a random value drawn from a Poisson distribution having a mean equal to the data value (as for counting statistics). For datasets with Gaussian noise, a random value drawn from a Gaussian distribution of mean zero and standard deviation $\sigma_{\text{noise}}$ was added to each data point. The signal to noise ratio (SNR) of the data was defined as the mean value of the signal within the area of the Gaussian peak, divided by the standard deviation of the noise. Specifically, the area of the peak was approximated as the area of a circle with a diameter equal to the full-width at tenth maximum (FWTM) of the Gaussian, i.e. $\text{Area}_{\text{Gaussian}}\approx\pi\left(\sigma\sqrt{2\ln10}\right)^{2}$. The volume of the Gaussian can be written as $2\pi A\sigma^{2}$, and hence the mean value of the signal is approximately $\left\langle f_{\text{Gaussian}}\right\rangle=A/\left(\ln10\right)$. Thus, the signal to noise ratio was defined as shown in equation .

$$\text{SNR}=\frac{A}{\sigma_{\text{noise}}\ln10}$$

We note that the constant baseline level $b$ does not enter into these expressions, since the absolute value of the baseline has no effect on the tests in which the SNR was varied.

For the experiment comparing the LSE and MLE estimators, both unweighted and weighted least squares fits were tested. For the weighted fit, the weights input of Gpufit was set equal to the inverse of the data values, with a maximum weight value of 1.0.

## Test parameters

The specific parameters used for each test are listed in Supplementary Table S1. These values define the number of fits in the test dataset, the noise added to the data, the parameters of the simulated Gaussians, the randomness of the initial guesses, and the data size (the size of each fit).

## Test outputs

The test results included the fit speed and fit precision. The execution time of each fit function was determined by measuring the total time taken for the Matlab call to the fit function (the mex function call) using the Matlab functions tic and toc. The fit speed was defined as the number of fits per function call divided by the execution time (resulting in a speed value measured in number of fits per second). A measure of the fit precision was obtained by calculating the absolute precision of the *x*-coordinate fit parameter (the standard deviation of the *x*-coordinate fit errors). For code profiling results shown in Fig. 3 and Supplementary Fig. S5 and S6, the execution times of individual sections of the code were measured using the C++ timing class std::chrono::high_resolution_clock and with CUDA cudaDeviceSynchronize() at the end of each CUDA kernel.

# STORM experiment

## Microscope and sample preparation

The STORM microscope, data analysis, and sample preparation procedures have been described previously.^3,4^ For this experiment, the sample was stained with a fluorescent antibody against the protein GP210, a component of the Nuclear Pore Complex (NPC), as described previously.^5^ The secondary antibody was, in this case, labeled with the fluorescent dye Alexa Fluor 647.

# Supplementary Figure S1: GPU processing example

| 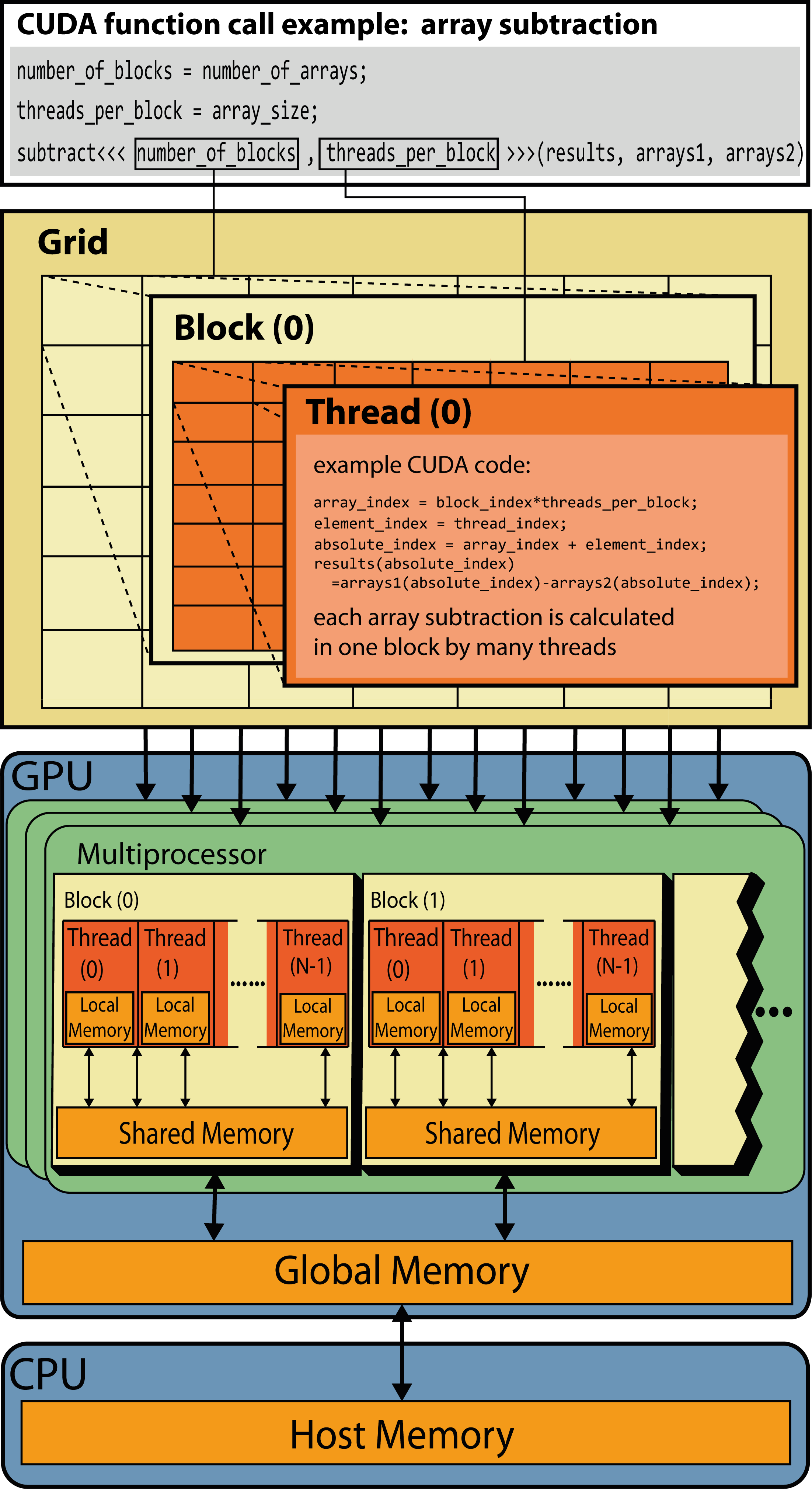 |
| --- |
| Supplementary Figure S1: GPU architecture and example computation. The GPU is organized into a set of multiprocessors. Each multiprocessor is logically organized into a grid of computing blocks, and each block contains multiple computing threads. The blocks have a dedicated shared memory space and may access global GPU memory. The example CUDA function shown here calculates the numerical difference between two sets of arrays. The input variables arrays1 and arrays2 each contain a set of M sub-arrays of N elements in length, concatenated together. The computation is spread across multiple blocks. Each array difference is calculated by one block. The difference between individual elements of the two arrays is calculated by individual threads. The number of blocks used in the calculation is equal to the number of input array pairs (M) and the number of threads per block is equal to the number of elements per array (N). As long as a sufficient number of computing blocks and threads are available, the entire calculation can be performed in a single step. |

# Supplementary Figure S2: Gpufit modular design

| 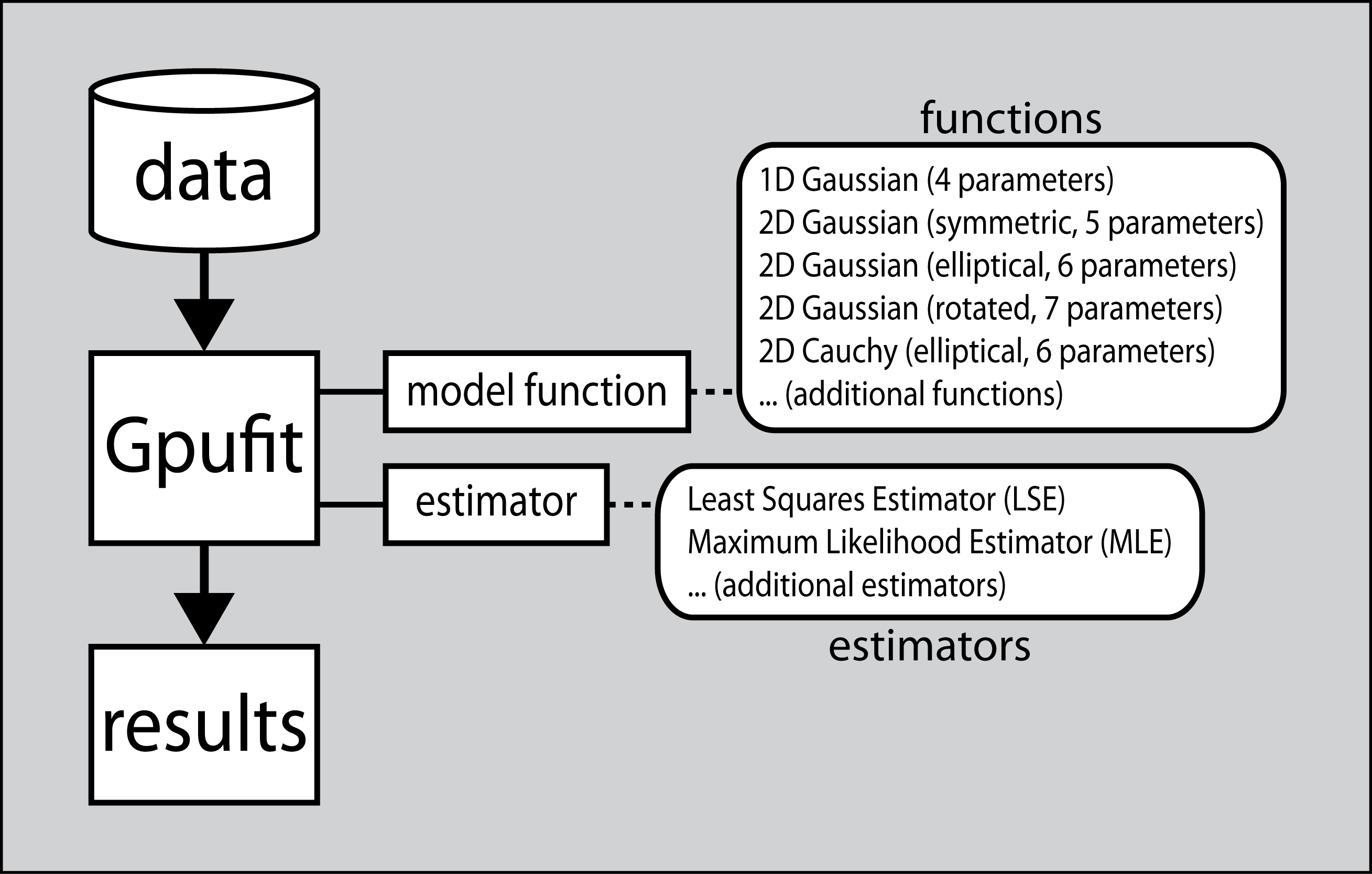 |
| --- |
| Supplementary Figure S2: The modular design of Gpufit. Data is transferred to the GPU in chunks of a suitable size. The core Gpufit module performs the fitting operation, making use of the selected model function and estimator, as specified by the user. Additional model functions and estimators may be added by re-compiling the source code. Finally, the results of the fit process are returned to CPU memory. |

# Supplementary Figure S3: Gpufit vs. MINPACK comparison

| 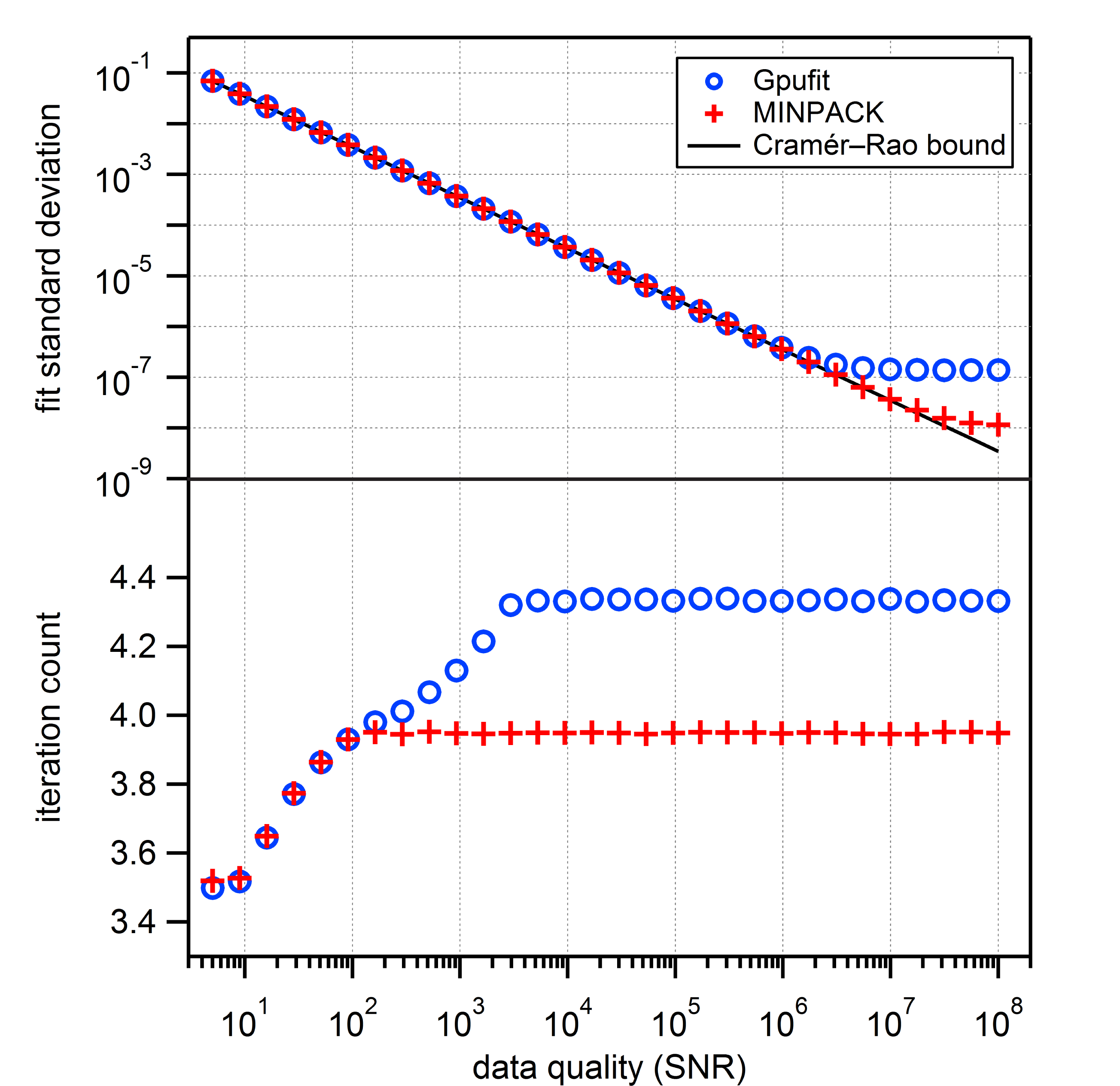 |
| --- |
| Supplementary Figure S3: Gpufit vs. MINPACK comparison. Upper plot shows the standard deviation of the fit results (absolute X position, arbitrary units) for a set of 2D Gaussian fits as a function of the signal to noise ratio (SNR) of the input data, for the two algorithms Gpufit and MINPACK. Similar results were obtained over a wide range of SNR values. For reference, the Cramér–Rao bound^6^ of the position estimator is also shown (black line), verifying that the fit results from both packages approach the theoretical limit. The lower plot shows the mean number of fit iterations required for convergence. For most conditions tested, MINPACK was found to converge in fewer iterations than Gpufit, as expected since MINPACK employs a modified version of the LMA. |

# Supplementary Figure S4: Fit precision and accuracy

| 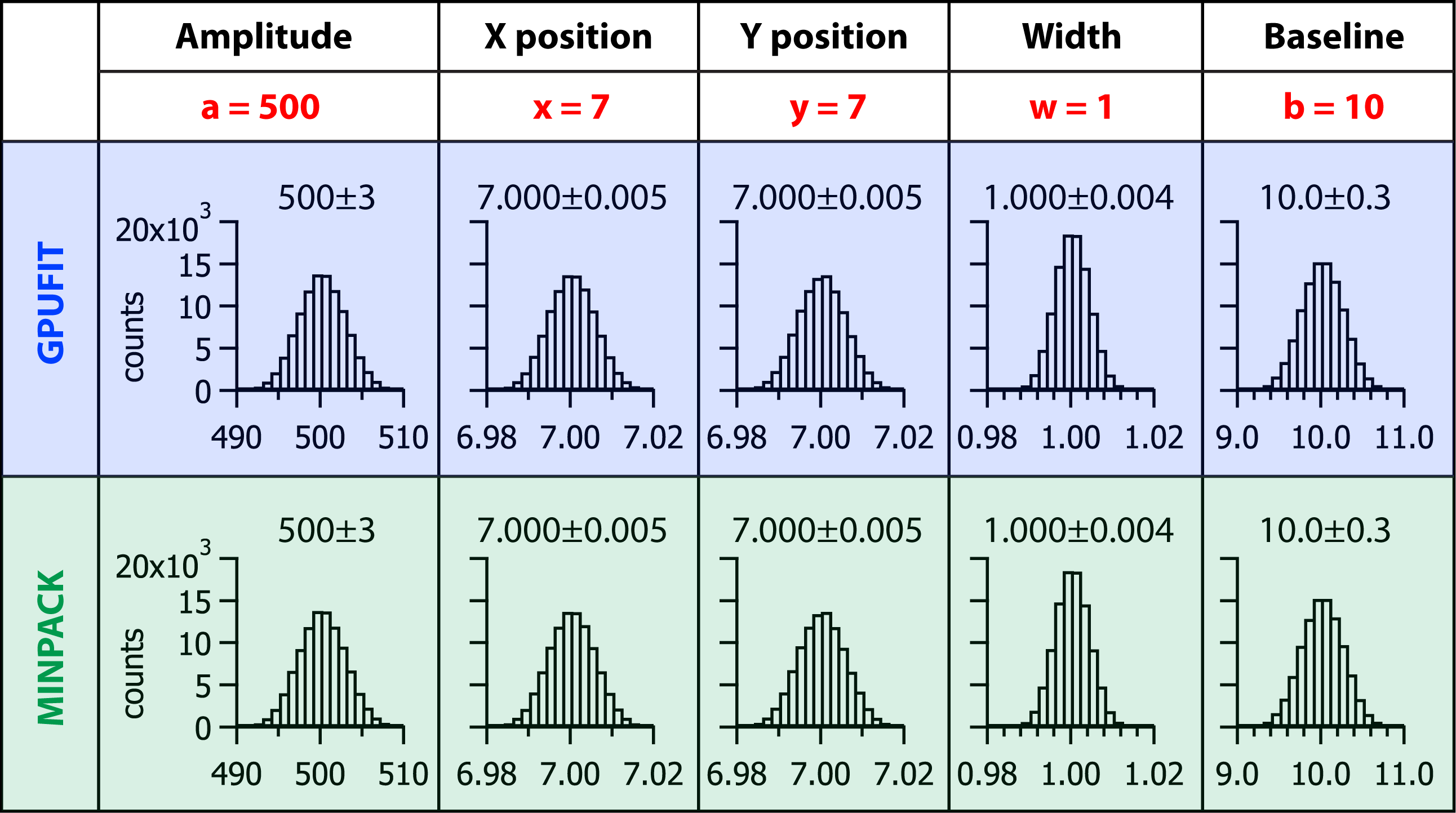 |
| --- |
| Supplementary Figure S4: Fit precision and accuracy comparison for Gpufit and Minpack. The model function is a five-parameter 2D Gaussian fit, and 10^4^ individual fits were calculated for a randomized dataset with a SNR of 100. The true values of each parameter are shown in red at the top of the figure, and below them the distributions of the fit results are plotted, along with the mean and standard deviation for each result. When comparing the results of Gpufit and MINPACK, no deviations were measured in fit precision or accuracy, with the exception of the results for very high SNR values as shown in Supplementary Fig. S3. |

# Supplementary Figure S5: Cpufit program flow

| 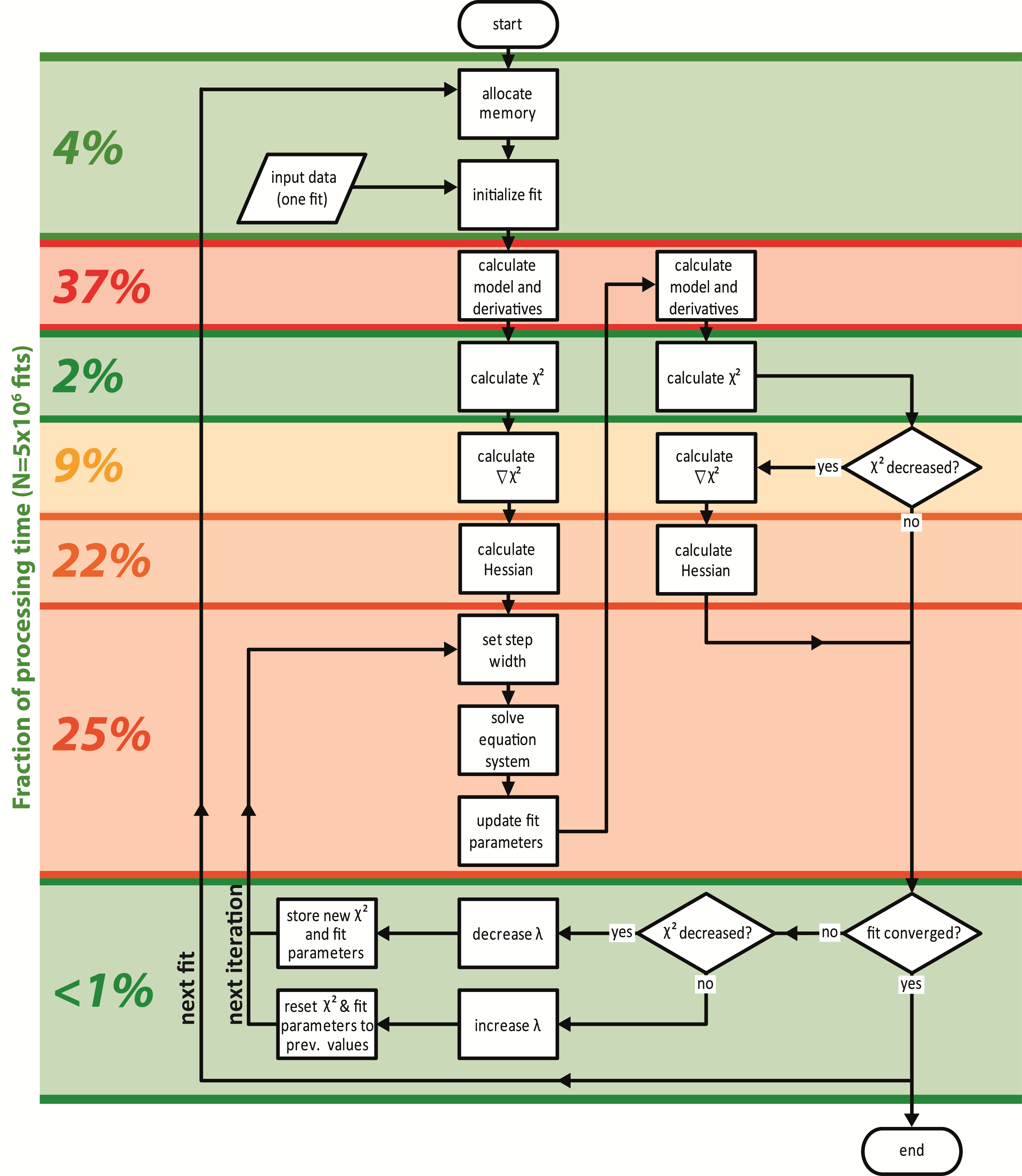 |
| --- |
| Supplementary Figure S5: Program flow and normalized execution times of Cpufit. Code profiling results for a sequence of five million individual fitting operations are shown on the left side of the chart (percentage of total time spent in each code section). The profiling results show that the majority of the execution time is spent calculating the model function at each data point, evaluating the Hessian matrix, and solving the equation system. |

# Supplementary Figure S6: Gpufit program flow

| 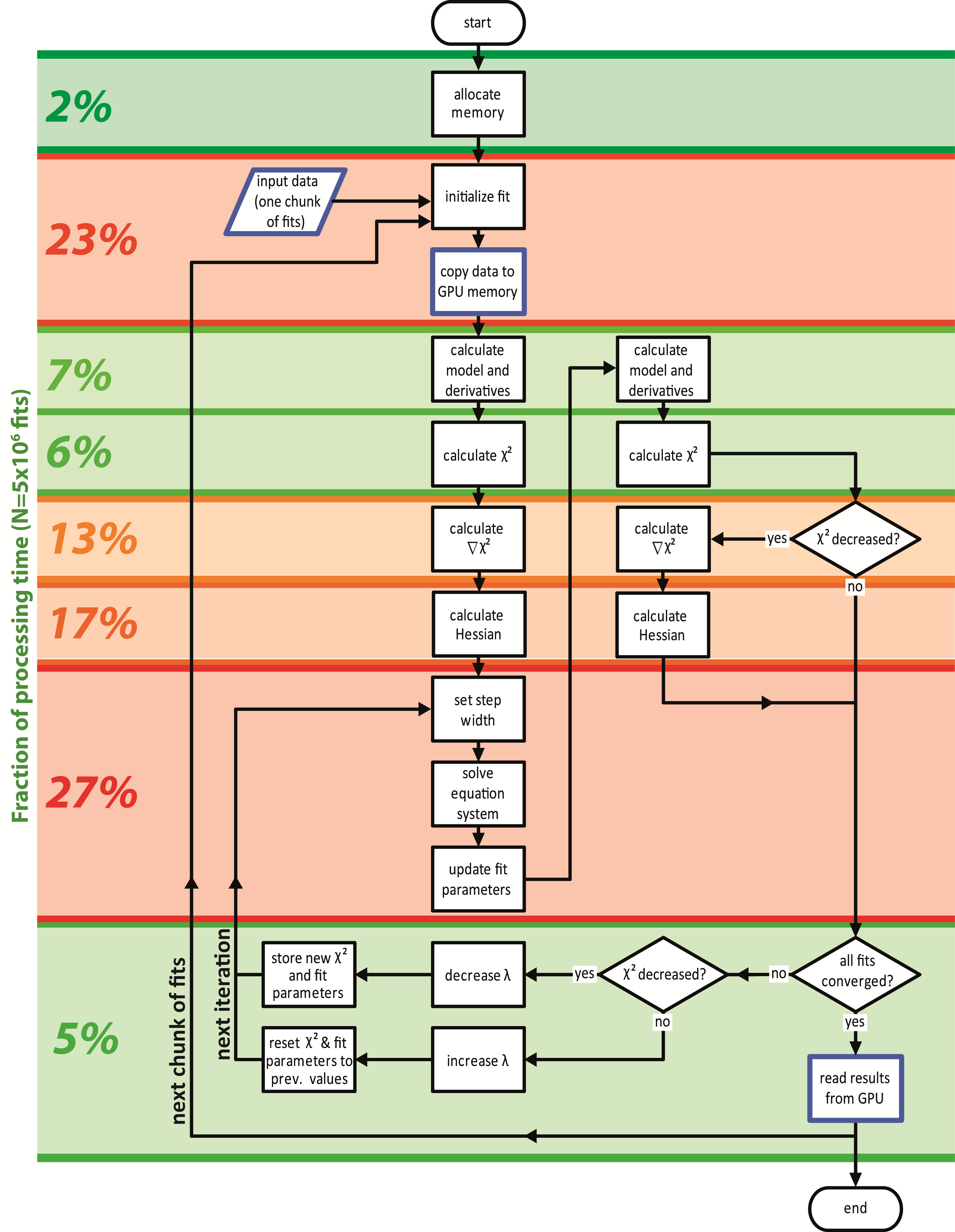 |
| --- |
| Supplementary Figure S6: Program flow and normalized execution times of Gpufit. Code profiling results for a sequence of five million individual fitting operations are shown on the left side of the chart (percentage of total time spent in code section). Additional and modified steps, as compared with the Cpufit program flow, are highlighted in blue. The profiling results show that data transfers to the GPU memory constitute a significant amount of the processing time, while tasks which are highly parallelized, such as the calculation of the model function, are relatively fast in comparison with the Cpufit program flow (see Supplementary Figure S5). Note that the program flow corresponds to one fit only, however many fits are processed in parallel. Once an individual fit has converged, it is not iterated further. |

# Supplementary Figure S7: Precision of Gpufit and GPU-LMfit

| 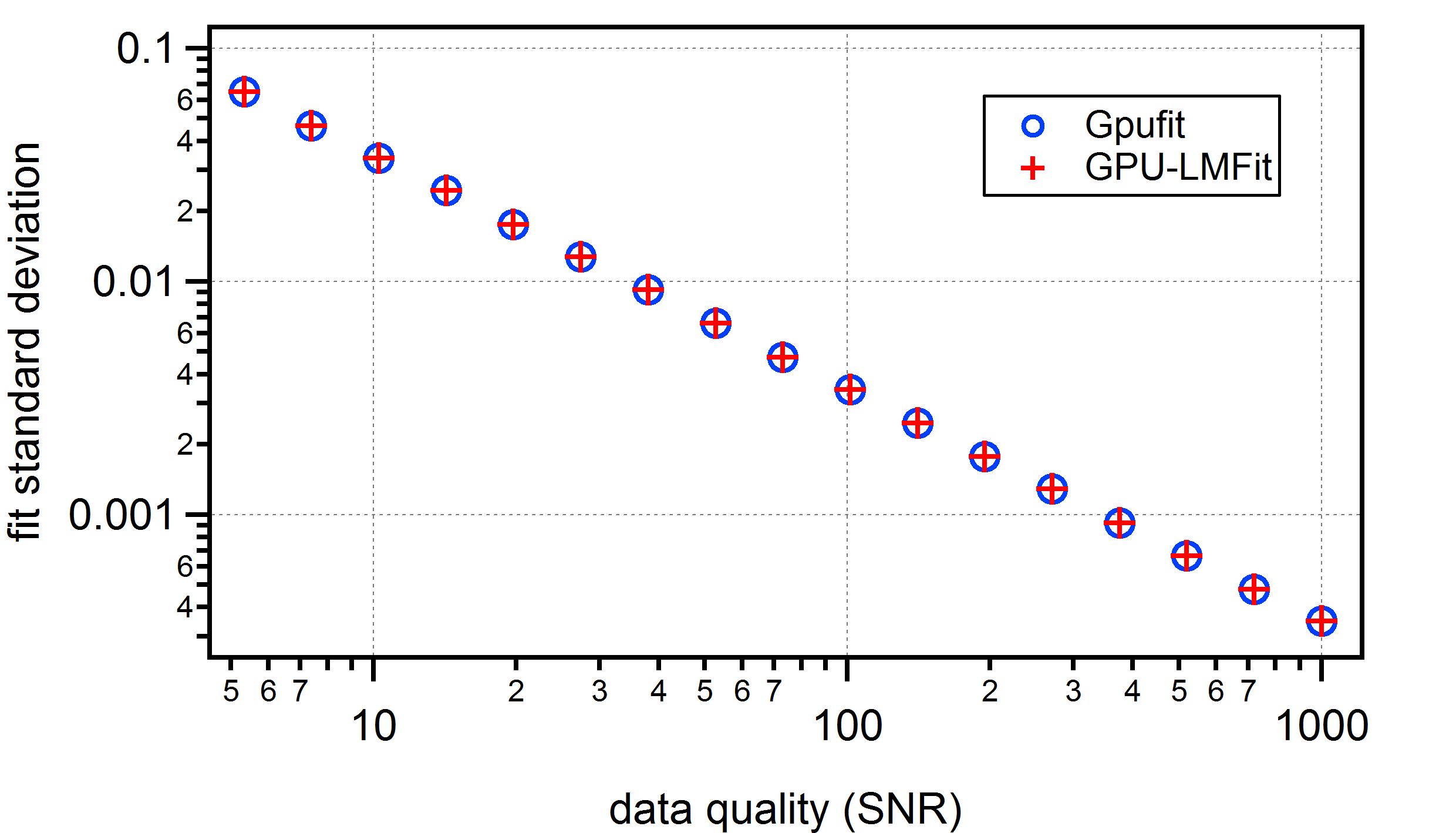 |
| --- |
| Supplementary Figure S7: Comparison of fit precision between two GPU-accelerated Levenberg Marquardt algorithms: Gpufit and GPU-LMFit. The absolute precision (standard deviation) of the x-position parameter (arbitrary units) returned by the fit algorithm is plotted on the vertical axis. As the SNR of the input data was varied, the two packages yielded results with virtually identical degrees of precision. |

# Supplementary Figure S8: Comparison of fit estimators

| 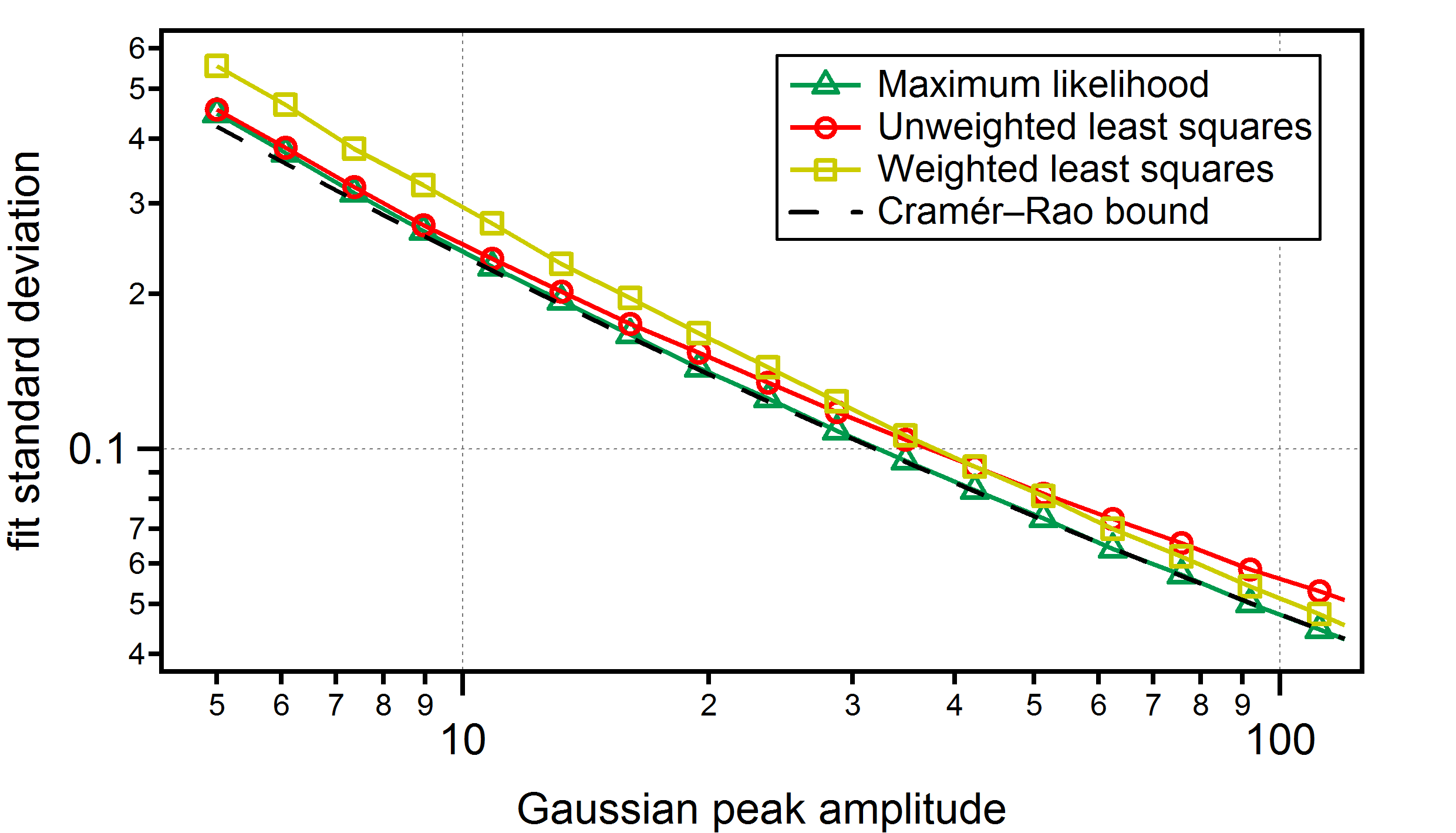 |
| --- |
| Supplementary Figure S8: Fit precision comparison for weighted and unweighted least squares vs. maximum likelihood estimation. For reference, the Cramér–Rao bound^6^ of the position estimator is also shown (dashed black line). The input data consisted of simulated 2D Gaussian functions with Poisson distributed (counting) noise. The signal to noise ratio of the data was varied by changing the amplitude of the Gaussian peak. The standard deviation of the fit results for the x-position parameter (arbitrary units) is plotted on the vertical axis. As expected, a more precise fit is obtained using the maximum likelihood estimator, as opposed to least squares fitting. For low signal levels, the unweighted least squares fit performed similarly to the maximum likelihood estimator, while for larger values a weighted least squares fit was comparable to the maximum likelihood fit. For all conditions tested, however, the maximum likelihood estimator yielded the highest fit precision. |

|  | Figure 1 | Figure 2 | Figure 3A | Figure 3B | Figure S3 | Figure S4 | Figure S5 | Figure S6 | Figure S7 | Figure S8 |
| --- | --- | --- | --- | --- | --- | --- | --- | --- | --- | --- |
| OS architecture | 64-bit | 64-bit | 32-bit | 32-bit | 64-bit | 32-bit | 64-bit | 64-bit | 32-bit | 64-bit |
| Number of fits per  function call (*N*) | varied:  10 to 10^8^ | 5.0 x 10^6^ | varied:  1 to 10^6^ | 1.0 x 10^5^ | 2.0 x 10^4^ | 1.0 x 10^5^ | 5.0 x 10^6^ | 5.0 x 10^6^ | 1.0 x 10^4^ | 1.0 x 10^4^ |
| Maximum # iterations | 20 | 20 | 20 | 20 | 20 | 20 | 20 | 20 | 20 | 20 |
| Fit tolerance | 1.0 x 10^-4^ | 1.0 x 10^-4^ | 1.0 x 10^-4^ | 1.0 x 10^-4^ | 1.0 x 10^-4^ | 1.0 x 10^-4^ | 1.0 x 10^-4^ | 1.0 x 10^-4^ | 1.0 x 10^-4^ | 1.0 x 10^-4^ |
| Fit weighting | None | None | None | None | None | None | None | None | None | Weighted and unweighted |
| Noise type | Gaussian | Gaussian | Gaussian | Gaussian | Gaussian | Gaussian | Gaussian | Gaussian | Gaussian | Poisson |
| SNR | 60 | 60 | 60 | 60 | varied: 5.0 to 10^8^ | 60 | 60 | 60 | varied:  5.0 to 10^3^ | --- |
| Data size (edge length *L*) | 5 | 5 | 5 | varied:  5 to 25 | 15 | 15 | 5 | 5 | 15 | 15 |
| Gauss amplitude (*A*) | 500 | 500 | 500 | 500 | 500 | 500 | 500 | 500 | 500 | varied:  5 to 200 |
| Gauss x position (*x_0_*) | center | center | center | center | center | center | center | center | center | center |
| Gauss y position (*y_0_*) | center | center | center | center | center | center | center | center | center | center |
| Gauss width (*σ*) | 1.0 | 1.0 | 1.0 | $0.143\times L$ | 1.0 | 1.0 | 1.0 | 1.0 | 1.0 | 2.0 |
| Gaussian baseline (*b*) | 10.0 | 10.0 | 10.0 | 10.0 | 10.0 | 10.0 | 10.0 | 10.0 | 10.0 | 5.0 |
| Coordinate deviation max. (*Δx_max_, Δy_max_*) | 0.5 | 1.0 | 0.5 | 0.5 | 1.0 | 0.0 | 1.0 | 1.0 | 1.0 | 1.0 |
| Initial guess max. offset fraction (*f_offse_*_t_) | 0.3 | 0.3 | 0.3 | 0.3 | 0.3 | 0.3 | 0.3 | 0.3 | 0.3 | 0.3 |

# Supplementary Table S1: Test parameters

Supplementary Table 1: The parameters which define the test datasets used for characterization of Gpufit performance. Parameters are ordered according to the figure in which the test results are shown.

# References

1 Du, P. *et al.* From CUDA to OpenCL: Towards a performance-portable solution for multi-platform GPU programming. *Parallel Comput.* **38**, 391-407 (2012).

2 Zhu, X. & Zhang, D. Efficient Parallel Levenberg-Marquardt Model Fitting towards Real-Time Automated Parametric Imaging Microscopy. *PLoS ONE* **8**, e76665 (2013).

3 Bates, M., Jones, S. A. & Zhuang, X. in *Imaging: A Laboratory Manual* (ed R. Yuste) 547-576 (Cold Spring Harbor Laboratory Press, 2011).

4 Bates, M., Dempsey, G. T., Chen, K. H. & Zhuang, X. Multicolor Super-Resolution Fluorescence Imaging via Multi-Parameter Fluorophore Detection. *ChemPhysChem* **13**, 99-107 (2012).

5 Göttfert, F. *et al.* Coaligned Dual-Channel STED Nanoscopy and Molecular Diffusion Analysis at 20 nm Resolution. *Biophys. J.* **105**, L01-L03 (2013).

6 Cramér, H. *Mathematical methods of statistics*. (Princeton University Press, 1946).
